# Mitochondrial heterogeneity and adaptations to cellular needs

**DOI:** 10.1038/s41556-024-01410-1

**Published:** 2024-05-16

**Authors:** Melia Granath-Panelo, Shingo Kajimura

**Affiliations:** 1 Division of Endocrinology, Beth Israel Deaconess Medical Center, Harvard Medical School and Howard Hughes Medical Institute, Boston, MA, USA.; 2 Department of Molecular Metabolism, Harvard T.H. Chan School of Public Health, Boston, MA, USA.

## Abstract

Although it is well described that mitochondria are at the epicentre of the energy demands of a cell, it is becoming important to consider how each cell tailors its mitochondrial composition and functions to suit its particular needs beyond ATP production. Here we provide insight into mitochondrial heterogeneity throughout development as well as in tissues with specific energy demands and discuss how mitochondrial malleability contributes to cell fate determination and tissue remodelling.

The energy demands of a cell vary according to cell type. As an energy hub, mitochondria are the site of aerobic ATP production, lipid synthesis, iron–sulfur cluster biogenesis and amino acid production; they are also involved in the conversion of intermediates of many essential metabolic pathways^1^. Although mitochondria are central to the homeostatic energy balance of the cell, fuel choices are often distinct between cell types (Box 1). For instance, substrate availability can govern cell fate and progenitor cell plasticity, and underlie cell identity^2^. The mitochondria of a progenitor cell with a glycolytic phenotype that maintains self-renewal (for example, embryonic stem cells) utilize glucose-derived pyruvate to fuel the citric acid cycle (TCA) and generate carbon-rich building blocks to support proliferation. On the other hand, committed post-mitotic cells shift towards oxidative phosphorylation (OXPHOS) and increase mitochondrial biogenesis^2^. Through the lens of an immune cell, mitochondria support rapid maturation and proliferation^3^. When considering the specific needs of a neuron, mitochondria are tailored to support branching and high signalling activity^4^.

Such cell-specific roles are supported, in part, by unique protein compositions of mitochondria. The best studied example is uncoupling protein 1 (UCP1)—a mitochondrial inner-membrane protein that is selectively expressed in brown and beige adipocytes. UCP1 is a mitochondrial proton carrier that uncouples the proton gradient from ATP synthesis, dissipating chemical energy in the form of heat, underlying the unique biological function of brown fat as a thermogenic organ in mammals^5^. Mitochondria in the liver express a unique set of proteins that are required for gluconeogenesis in response to fasting by exporting gluconeogenic substrates into the cytosol^6^. Mitochondrial complex I, or NADH dehydrogenase, is dispensable, demonstrating flexibility in the maintenance of the NAD^+^/NADH ratio for hepatocyte homeostasis^7^.

Given the influence of mitochondria on various cell behaviours, it is perhaps unsurprising that these functions can permeate to alter tissue-selective properties. In part, this is represented in how mitochondrial function ebbs and flows with the rhythm of developmental signals and cues, as reducing OXPHOS in the developing mesoderm and endoderm reduces differentiation efficiency and developmental rate^8^. Recent evidence postulates that the protein composition of mitochondria underlies cell identity long before organogenesis^9^. These observations are indicative of how mitochondrial function in a cell can influence tissue behaviour—both development and remodelling of mature tissues—and how varied mitochondrial composition and functions can be. In this Review we discuss mitochondrial adaptations to cellular contexts and how mitochondrial bioenergetics and fuel selection influence development and tissue-specific functions.

## Mitochondrial adaptations in progenitor cell plasticity

In mouse and human embryonic stem cells in culture, induction of differentiation is accompanied by decreased glycolytic activity, which ultimately results in decreased capacity for self-renewal^10-12^. This, along with similar observations in other stem cell populations, has led to the conclusion that glycolytic activity underlies the maintenance of stemness^13^. The stem cell niche is often a hypoxic environment in which cells rely mainly on anaerobic glycolysis as the main energy and fuel source. However, not all progenitor cell niches are hypoxic and oxygen concentration is an important determinant of stem cell maintenance^14,15^. Although glycolytic activity is generally preferential for the maintenance of mesenchymal and neural progenitor cells (NPCs), haematopoietic stem cells (HSCs), which are of mesodermal origin, have a comparable functional mitochondrial content to terminally differentiated cells^16-18^. Furthermore, the nutritional environment of commercially available media, in which glucose and glutamine are abundant, results in increased glycolytic activity and lipogenesis in human pluripotent stem cells, whereas cells in embryonic fibroblast-conditioned media have higher TCA-cycle turnover and OXPHOS activity^19^. Therefore, the glycolytic metabolism of stem cells is affected by the surrounding environment.

**Fig. 1 ∣:**
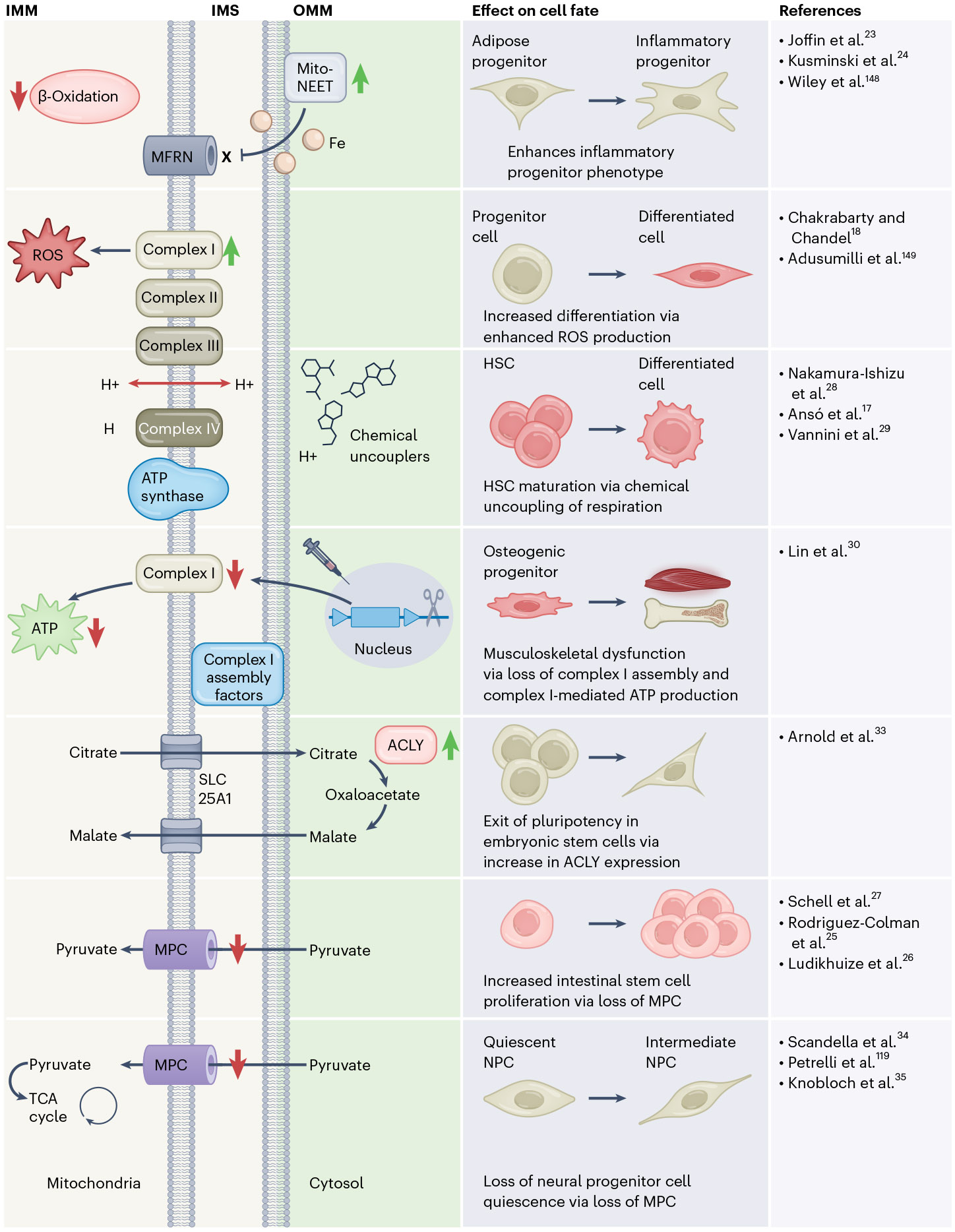
Changes in mitochondrial metabolism that drive progenitor cell behaviours. Inducible expression of MitoNEET, which prevents iron entry into mitochondria through MFRN (also known as SLC25A37) and subsequently reduces β-oxidative activity in pre-adipocytes, directs the adipose progenitor lineage to a pro-inflammatory state. An increase in mitochondrial complex I activity, and subsequently ROS production, negatively affects stem cell self-renewal and often results in enhanced cell maturation and differentiation. The addition of chemical uncouplers—such as carbonyl cyanide-*p*-trifluor omethoxyphenylhydrazone, which dissociates electron transfer from ATP production—results in maturation of HSCs. Loss of complex I assembly factors in the osteogenic progenitor, via inducible complex I assembly factor knockout, renders complex I inactive and subsequently drives a reduction of ATP production, leading to damaged skeletal progenitors. Engagement of non-canonical TCA cycle activity, whereby citrate is exported out of mitochondria to generate cytosolic OAA, underlies the embryonic stem cell exit from pluripotency. Loss of MPCs in intestinal stem cells results in maintenance of the stem cell pool as well as intestinal stem cell proliferation and self-renewal. Conversely, reduced pyruvate entry via MPC in NPCs can trigger loss of quiescence in the NPCs, resulting in the maturation to intermediate NPCs and eventually mature nerve cells. IMM, inner mitochondrial membrane; IMS, mitochondrial intermembrane space; and OMM, outer mitochondrial membrane. Refs. 148,149 are cited in this figure.

The contribution of mitochondria to the function and maintenance of many stem cell populations has been under investigation in recent years. For instance, adipose progenitor cells have low mitochondrial activity and subscribe to the highly glycolytic model of energy utilization; however, when progressing through lineage commitment and differentiation, they increase their mitochondrial content and activity^20,21^. Lineage-committed beige adipocyte progenitor cells possess cristae-dense mitochondria and high levels of fatty acid oxidation under a proliferative state, such as exposure to cold^22^. On the other hand, reduction of the mitochondrial oxidative capacity of adipose progenitor cells promotes pro-inflammatory responses and limits the lineage commitment to adipocytes^23,24^. As an example, adipose-specific overexpression of a singular outer mitochondrial membrane protein, MitoNEET, which inhibits iron entry into the mitochondria, limits β-oxidative capacity and promotes an inflammatory progenitor state^23^ (Fig. 1). Thus, mitochondrial metabolism profoundly influences adipocyte progenitor cell growth and lineage commitment. In the intestinal epithelium, where there is a high rate of cellular turnover, perturbation of glycolysis via the addition of dichloroacetate or 2-deoxy-D-glucose transitions intestinal stem cells towards OXPHOS and drives intestinal crypt formation^25^. A decrease in mitochondrial respiration promotes differentiation of intestinal crypt cells to secretory Paneth cells, and the mitochondrial activity of intestinal stem cells is connected to cell adhesion signalling through a Notch–FOXO axis^26^. Intestinal stem cells maintain a glycolytic reliance while still keeping a high and functional mitochondrial content^27^.

HSCs sustain life-long haematopoiesis and exist well into adulthood. Dormant HSCs exist in a hypoxic stem cell niche that promotes a glycolytic phenotype while still maintaining detectable mitochondrial activity^28^. Despite mainly relying on glycolysis, complete disruption of respiration in HSCs impairs haematopoiesis and HSC functions^17^. However, chemical uncoupling of respiration diverts HSCs towards rapid proliferation in differentiation conditions, suggesting that fate decisions in HSCs can be made as a result of altered mitochondrial membrane potential^29^. Disruption of mitochondrial complex I assembly in skeletal progenitors, which can give rise to bone-forming osteoblasts, impairs osteogenesis and causes musculoskeletal defects^30^. Although stem and progenitor cells generally prefer glucose as their main fuel source, the presence of functional mitochondria—in amounts that are similar to differentiated cells in certain cell types—remains essential for self-renewal and overall progenitor cell viability. Disruption of respiration in these predominantly glycolytic cells results in an inability to differentiate, alluding to the fact that proper levels of OXPHOS activity, depending on the cell type, are still important for general progenitor cell health^31^.

Although progenitor cells can divert pyruvate away from mitochondrial OXPHOS by way of pyruvate carboxylase, the TCA cycle dependency can be further nuanced. TCA cycle intermediates, including citrate and α-ketoglutaric acid, can act as retrograde signalling molecules that control the transcription and epigenetic processes in the nucleus, altering stem cell fate^18,32^. This phenomenon could also explain why progenitor cells have a large presence of functional mitochondria. Embryonic stem cells undergoing cell fate transition engage in a non-canonical TCA cycle by which oxaloacetate (OAA) is regenerated in the cytosol via mitochondria-derived citrate^33^. Using [U-^13^C] glucose tracing to monitor TCA cycle activity in embryonic stem cells, this mechanism was shown to be dependent on ATP-citrate lyase (ACLY) and an increase in ACLY expression resulted in an exit of naive pluripotency and a corresponding reliance on this non-canonical TCA cycle metabolism^33^. Intestinal stem cells express low levels of the mitochondrial pyruvate carrier (MPC), and changes in MPC expression selectively control intestinal stem cell fate^27^. Analysis of single-cell transcriptomic and proteomic datasets of neural stem cells revealed that quiescent adult NPCs have high expression levels of TCA-cycle components^34^. Glucose-derived pyruvate, which in turn fuels the TCA cycle, is required to maintain NPCs in a quiescent state^35^. Although they clearly have different forms and functions, it is interesting to compare the low expression of the MPC in highly proliferative intestinal stem cells with the high MPC expression in the non-proliferative quiescent NPCs, as neural stem cells seem to require basal levels of OXPHOS activity and oxygen consumption to maintain quiescence and hinder proliferation in contrast to intestinal stem cells.

## Mitochondrial network changes in development

### Mitochondrial adaptations in development

Changes in mitochondrial protein composition, ultrastructure, oxidative ATP production capacity and overall content occur during development, furthering the branching of cell lineages^9,12^. Recent work postulates that distinct mitochondrial protein composition underlies cell identity in early organogenesis^9^. In addition to organelle composition, specific mitochondrial localization allows for myofibroblast motility and subsequent alveolar development in the lung^36^. In the brain, changes in mitochondrial location and ultrastructure can affect circuit development, dendritogenesis and axon growth^37^. In the cardiovascular system, fine-tuned mitochondrial function is critical for proper development of the heart. A preference for anaerobic glycolysis in cardiomyocytes has been observed despite evidence of coupled OXPHOS activity and ATP production^38^. This metabolic flexibility allows for proper development that is suited to the conditions of the organism. Although these perturbations occur during organ-specific development, deletion of mitochondrial ribosomal proteins results in a complete failure to initiate gastrulation in the early embryo, demonstrating the importance of functional mitochondria during early development^39^. This poses interesting questions as to (1) when exactly the changes in mitochondrial network, organization, composition and activity are occurring, and (2) whether mitochondrial behaviours are pre-determined before cell lineage commitment and drive fate determination.

The oocyte is a unique cell type formed during early fetal development that remains viable for many years^40^. To maintain viability, oocytes are distinctly energetically demanding while needing to maintain a dormant state; defects in mitochondrial function can result in defective oocyte maturation and quality, and negatively impact fertility^41^. Like most cells, oocytes are reliant on a careful balance of the right amount of reactive oxygen species (ROS)—a sweet spot of enough ROS for normal oocyte processes such as meiotic division and functioning as second messengers for maturation but not enough to cause damage^42^. Oocytes remain dormant by shutting down mitochondrial complex I activity while keeping the rest of the electron transport chain (ETC) active^40^. This allows for the synthesis of essential molecules to support oocyte health for the long term while keeping complex I-driven ROS production at bay^40^.

### Mitochondrial control of developmental rates

During early embryonic development and throughout organogenesis, mitochondria undergo dynamic morphological changes to support specific functions. At first, their morphology is mainly spherical, with shortened and unorganized cristae, as opposed to elongated and ordered as seen in differentiated cells^43^. The early embryo needs to maintain low ROS production while still producing macromolecules for maturation, so having a spherical mitochondrial ultrastructure could be advantageous, as increasing the cristae surface area could give way to enhanced ETC activity and ROS-mediated signalling^44^. As tissue specificity is established, the mitochondrial network becomes more elongated and linear with an organized cristae structure^2^. In human cortical neural progenitors, mitochondria in pre-mitotic as well as actively mitotic cells are fragmented. By contrast, mitochondria are increased in size and quantity in post-mitotic mature cells, suggesting a post-mitotic mechanism by which mitochondrial network development influences neurogenesis^45^. In the developing brain, glucose-derived pyruvate is a necessary component to support processes such as synapse formation and axon growth^46^. Interestingly, the rates of change in mitochondrial ultrastructure differ between mouse and human cortical neurons: mitochondrial oxygen consumption and TCA cycle activity are lower in human neurons compared with mouse neurons and an increase in mitochondrial activity accelerates human neuronal maturation^45^.

In an induced pluripotent stem cell-derived model of the presomatic mesoderm, the impairment of OXPHOS-linked respiration via manipulation of the cellular NAD^+^/NADH redox balance lowers the protein synthesis rate and slows developmental cues in this system^8^. An increase in the NAD^+^/NADH ratio via overexpression of *Lb*NOX, an engineered bacterial NADH oxidase, increases protein synthesis and the overall developmental rate, and these differences are recapitulated in both mouse and human three-dimensional cell models^8^. Mitochondrial–nuclear crosstalk determines tissue-specific mitochondrial composition before the initiation of organ maturation in the developing embryo, suggesting that mitochondrial composition precedes cell identity^9^. The distinct composition of these mitochondria is determined via nuclear-encoded genes that are regulated transcriptionally, which reflect lineage-specific transcriptional signatures observed in mature cell types^9^. In a similar line to mitochondrial–nuclear crosstalk, mitochondria-localizing enzymes that contribute to TCA cycle transiently translocate to the nucleus at the zygotic genome activation stage of the early mouse embryo to induce epigenetic remodelling^47^. Although these studies provide insight into how mitochondrial function can drive early embryonic germ layer development, further work is required to test the central roles that mitochondria play in early organ-specific development, to dissect the mechanisms underlying customized mitochondrial composition and metabolic programmes and to analyse how changes in mitochondrial network relate to the environment and fate of a cell.

## The role of mitochondria in tissue remodelling

### Adipose tissue remodelling

The adipose tissue is a highly heterogenous organ that contains adipocytes (parenchymal cells), tissue macrophages, pre-adipocytes, fibroblasts, the neuronal and vascular architecture as well as adipose progenitor cells. Each cell has its own function within the tissue, yet their functional properties can vary from depot to depot^48^. Adipose-tissues undergo rapid adaptative remodelling in response to both healthy and pathological stimuli.

**Fig. 2 ∣:**
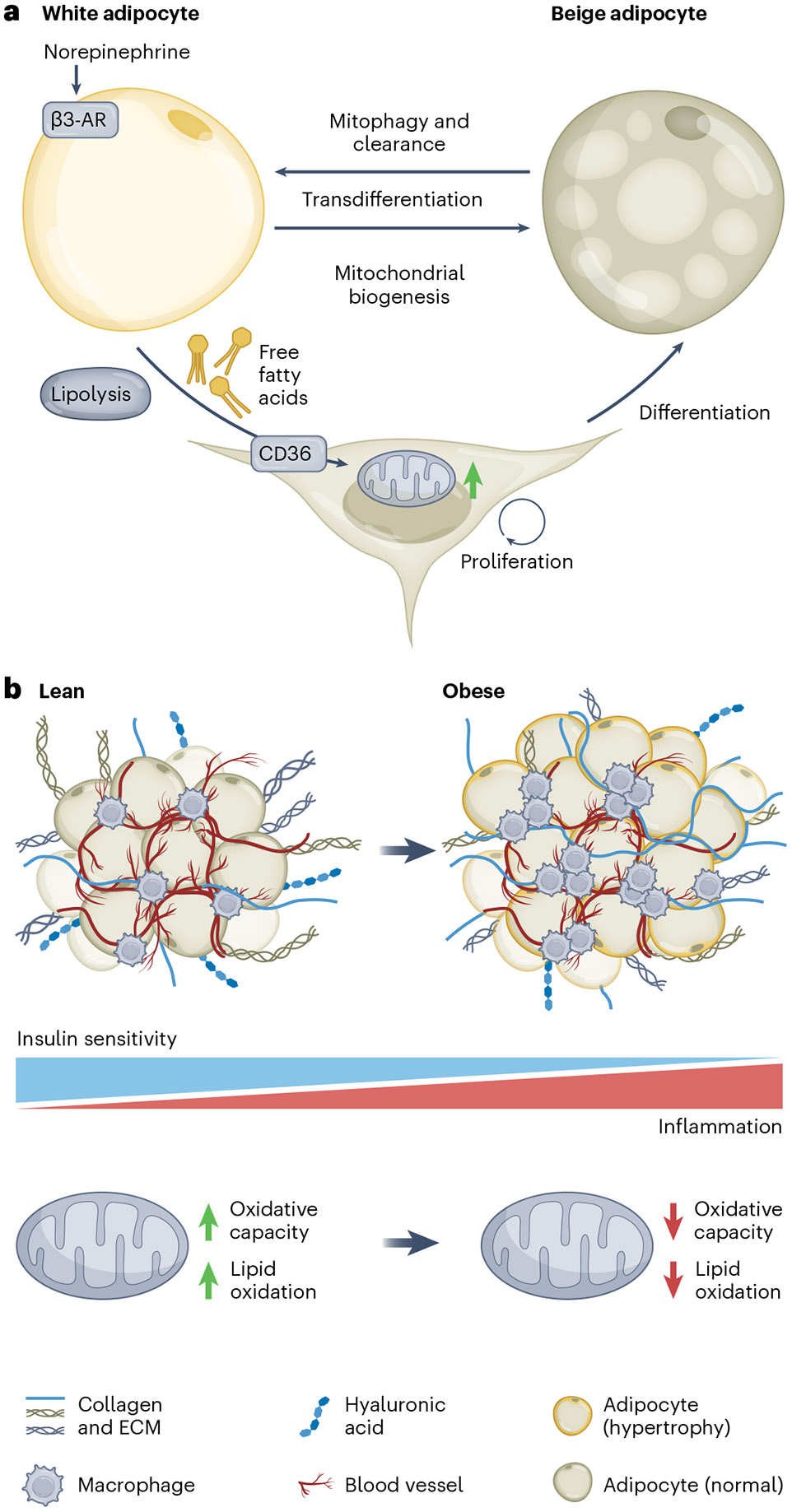
Adipocyte plasticity in tissue remodelling. **a**, Beige progenitor cell proliferation, differentiation and beige-to-white fat conversion. Stimulation of the β-3 adrenergic receptor (β3-AR) via norepinephrine results in lipolysis and release of free fatty acids, which signal to adipose progenitors via internalization by CD36. This drives proliferation and mitochondrial biogenesis of beige progenitors and subsequent differentiation to beige adipocytes. White adipocytes can transdifferentiate to beige via factors that induce mitochondrial biogenesis. Beige adipocytes can undergo transdifferentiation to white via mitophagy-mediated clearance of mitochondria. **b**, Adipose tissue of lean individuals is more insulin-sensitive than adipose of obese individuals. Adipose of obese individuals has a more inflammatory phenotype, with increased macrophage recruitment and subsequent stiff ECM deposition as a result of tissue hypoxia. Mitochondrial oxidative capacity and lipid oxidation is higher in lean individuals as opposed to obese individuals.

Physiologically healthy tissue undergoes remodelling in response to stimuli such as cold exposure and adrenergic stimuli^49^. Beige progenitor cells already possess mitochondria with high fatty-acid-oxidation capacity^22^. Following cold exposure and subsequent activation of adipose tissue lipolysis, beige progenitor cells take up fatty acids via the plasma membrane-fatty acid transporter CD36 and undergo cell proliferation and beige adipocyte differentiation—that is, de novo beige adipocyte biogenesis^50^ (Fig. 2a). A subset of differentiated adipocytes present a beige adipocyte phenotype through the activation of β-3 adrenergic receptor signalling and the regulation of mitochondrial dynamics^51^. For instance, ubiquitous overexpression of a mitochondrial cristae biogenesis protein, optic atrophy 1 (encoded by *Opa1*), induces beige adipocyte biogenesis as well as improved glucose tolerance and insulin sensitivity^52^. Conversely, mitochondrial clearance by mitophagy promotes the direct conversion from beige adipocytes to white adipocytes^53^. Reduced autophagy flux through the deletion of ATG5 or ATG12 promotes the retention of beige adipocytes and maintains high thermogenic capacity^54^.

During the onset of obesity, adipocytes expand in size (hypertrophy) due to the increase in lipid uptake^55^ (Fig. 2b). With this expansion in adipocyte size comes an induction of tissue hypoxia and a responsive upregulation of hypoxia-inducible factor-1α (HIF-1α)^56^. Furthermore, the increased free fatty acid uptake that is observed in obesity as a storage mechanism decreases oxidative capacity^57^. Elevated fatty acids activate SLC25A5, the mitochondrial ADP/ATP carrier, and also increase oxygen demand and drive tissue hypoxia and fibrotic remodelling^58^. Deletion of SLC25A5 elevates tolerance to hypoxia by reducing the oxygen demand of adipocytes while still keeping mitochondrial form and function intact^59^. Efforts to characterize other mediators of adipose dysfunction during the onset of obesity have identified the impairment of phosphocreatine metabolism in white adipose tissue^60^. Creatine kinase B catalyses the conversion of creatine to phosphocreatine, a rapidly accessible phosphate energy pool^61^. While phosphocreatine metabolism is impaired in obesity, adipocyte deletion of creatine kinase B increases the ATP:ADP ratio in adipocytes and promotes a pro-inflammatory state within adipose tissue, demonstrating the role of creatine kinase B as a mediator of pathophysiological adipose remodelling^60^. While there are distinct changes in adipocyte mitochondria during obesity-related adipose tissue remodelling, multiple adipose-resident cell types mediate the progression of these pathologies in conjunction with adipocytes.

Aside from adipocytes, macrophages adopt a distinct bioenergetic profile depending on the local tissue environment^62^. Mitochondrial dysfunction in adipose macrophages induces activation of the NLRP3 inflammasome, driving inflammation and subsequent fibrosis^63^. Macrophages from obese mice exhibit an activated bioenergetic profile and increased oxygen consumption, whereas macrophages from lean mice are more metabolically quiescent^62^. The relationship between macrophage function and adipose tissue health suggests therapeutic potential if the behaviour of the macrophage can be selectively leveraged. For instance, inhibition of macrophage activation by increasing OXPHOS activity via the ROS–AKT–ACLY pathway results in protection against diet-induced obesity and insulin resistance^64^. Part of the remodelling process in the development of obesity is an excess deposition of stiff extracellular matrix (ECM), which ultimately leads to tissue fibrosis^65^. Macrophages are a major producer of this ECM, and stiff ECM production is driven by HIF-1α signalling, promoting the synthesis of pro-fibrotic collagen species^56^. Compromise of mitochondrial respiration in otherwise healthy adipose progenitors promotes the development of fibrotic precursor cells and inflammation, which ultimately affects macrophage activation activity^23^. The pro-inflammatory state of adipose progenitors—which is driven by the extent of their β-oxidative capacity—activates macrophage recruitment to the adipose tissue and increases the amounts of macrophages in transitionary activation states^23^. More work is needed to understand how mitochondrial metabolism relates to recruitment and activation of resident adipose tissue macrophages in the context of the overall homeostasis of the adipose tissue.

### Connecting the mitochondria, the actin cytoskeleton network and the ECM

Mitochondria are now understood to be involved in the coordination of developmental signals and processes and, conversely, are involved in the remodelling of various mature tissues in response to varying stimuli. How are these organelles coordinating these processes? Emerging evidence highlights a connection between mitochondria, the actin cytoskeleton and the ECM that serves to transduce signals coordinating tissue and mitochondrial dynamics^66^. Actin can alter properties of the mitochondrial network and mitochondria can in turn affect the actin cytoskeleton. De novo F-actin assembly can occur on mitochondria and impaired DRP1-mediated mitochondrial fission results in prolonged F-actin accumulation time; in addition, disruption of actin polymerization via latrunculin B causes elongation of mitochondria^66,67^. In skeletal muscle, F-actin polymerization after plasma membrane injury depends on RhoA activity as a result of increased Ca^2+^ uptake via the protein mitochondrial calcium uniporter and subsequent OXPHOS-derived ROS^68^. Actin polymerization and assembly is regulated by three major classes of proteins, one of these proteins being Spire, which has been implicated in nuclear DNA repair and vesicle trafficking^69^. A mitochondrial form of Spire, from an alternative-splice variant of Spire1, links the mitochondria to the actin cytoskeletal network to drive mitochondrial fission^70^. Importantly, mitochondrial fission can alter autophagy, differentiation, organelle biogenesis and reliance on OXPHOS^71^. In this regard, a functional analysis of the effects of actin polymerization and mechanosensitive signalling on mitochondrial ultrastructure and function will be important.

In the chondrocyte, a post-mitotic cell type that remains viable for several years, low ECM turnover is required to maintain cell viability^72^. Loss of glycogen synthase kinase 3β activity results in increased mitochondrial oxidative damage, which increases ECM turnover and loss of healthy chondrocytes^72^. Dysfunction of OXPHOS in mature cartilage, which produces a large amount of ECM to maintain elasticity throughout life, increases collagen crosslinking and matrix stiffness, indicating bidirectional communication between the ECM and mitochondria^73^. As the ECM and associated proteins are involved in a variety of signalling processes, the next step will be to identify the signalling pathways that connect these two structures. For example, culturing cells on stiff versus soft substrate alters adhesion-mediated mechanosignalling through a β1 integrin signalling pathway, which involves ROCK-mediated activation of SLC9A1, and ultimately HSF1, and induces mitohormesis in response to a stiff ECM^74^. Other metabolic processes, such as glycolytic activity and beige adipogenesis, are responsive to changes in mechanosensitive signalling events such as substrate stiffness or actomyosin formation^75^. Although accumulating evidence demonstrates that signals from the ECM and actin cytoskeleton can affect mitochondrial function, the bidirectionality of these pathways is not well understood.

## Metabolic adaptation to bioenergetic demands

In energetically demanding tissues—such as the brain, skeletal muscle and the liver—the energy needs of the cell are constantly changing depending on the external stimuli. Efforts to characterize mitochondrial function across tissues have relied mainly on assessing the activity of proteins such as citrate synthase or measurement of mitochondrial DNA content, but these methods do not always reflect functional heterogeneity^72^. Omics-based profiling and functional assessment have identified quantitative differences in ubiquitously expressed mitochondrial proteins^4,76^. Importantly, these quantitative differences suggest fuel choice selectivity between cell and tissue types; how these enriched components across tissues relate to cell or tissue function is an area of ongoing investigation; a list of tissue-specific mitochondrial behaviours is provided in Table 1.

### The brain

The brain uses around 20% of the body’s energy and there is high regional specificity that gives different cell types a corresponding function^77^. Glucose is known to be the primary source of energy in the brain but there are differences in neuronal mitochondrial composition that dictate cell-specific function. There is high mitochondrial proteomic diversity across neural cell types in the cerebellum and this diversity has a functional purpose^4^. Astrocytes have high levels of β-oxidation machinery proteins, which alludes to a relationship between astrocytes and neurons—astrocytes soak up extra fatty acids via endocytosis of neuron-derived lipid particles to prevent fatty acid toxicity in neurons^78^. Astrocytes induce a transcriptional detoxification programme that supplies fatty acids to the mitochondria for β-oxidation in response to neuronal activity^78^. Although these behaviours make sense for the astrocyte given its support role, neurons are instead particularly vulnerable to oxidative- and fatty acid-induced damage^79^. The mitochondria in neurons are situated close to areas of high glucose concentration but how this affects neuronal mitochondrial function and substrate utilization is unclear^80^.

Profiling of mitochondrial heterogeneity has revealed differences in the enrichment of key mitochondrial programmes in different subcellular compartments in neurons. For example, components of the ETC are upregulated in mitochondria at synaptic terminals, as opposed to non-synaptic compartments^81^. Contrastingly, however, neurons may directly rely first on glycolytic activity for rapid energy, which would not require neuronal mitochondria or astrocytic glycolysis, followed by an increase in oxygen consumption, indicative of increased OXPHOS, evidenced by higher NADH production derived from glycolysis instead of NADH shuttling to mitochondria^82^. These described cell-type-specific enrichments reflect the role of each nerve cell type and their need for instant or sustained energy yet little is known about the contributions to energy production and consumption as a result of mitochondrial heterogeneity^80^. The expression of PGC-1α, which is linked to energy metabolism and mitochondrial biogenesis, is enriched in GABAergic inhibitory neurons and excitatory glutamatergic projection neurons, including medium spiny neurons^83^. Brain cells exhibit high fuel choice flexibility to maintain activity in periods of starvation^80,84^. Metabolite tracing shows that the preferential fuel for brain ATP production is generally glucose but ketone bodies can be utilized during fasting^83,85^. The mechanisms involved in this metabolic flexibility remain to be fully understood.

### Skeletal muscle

**Fig. 3 ∣:**
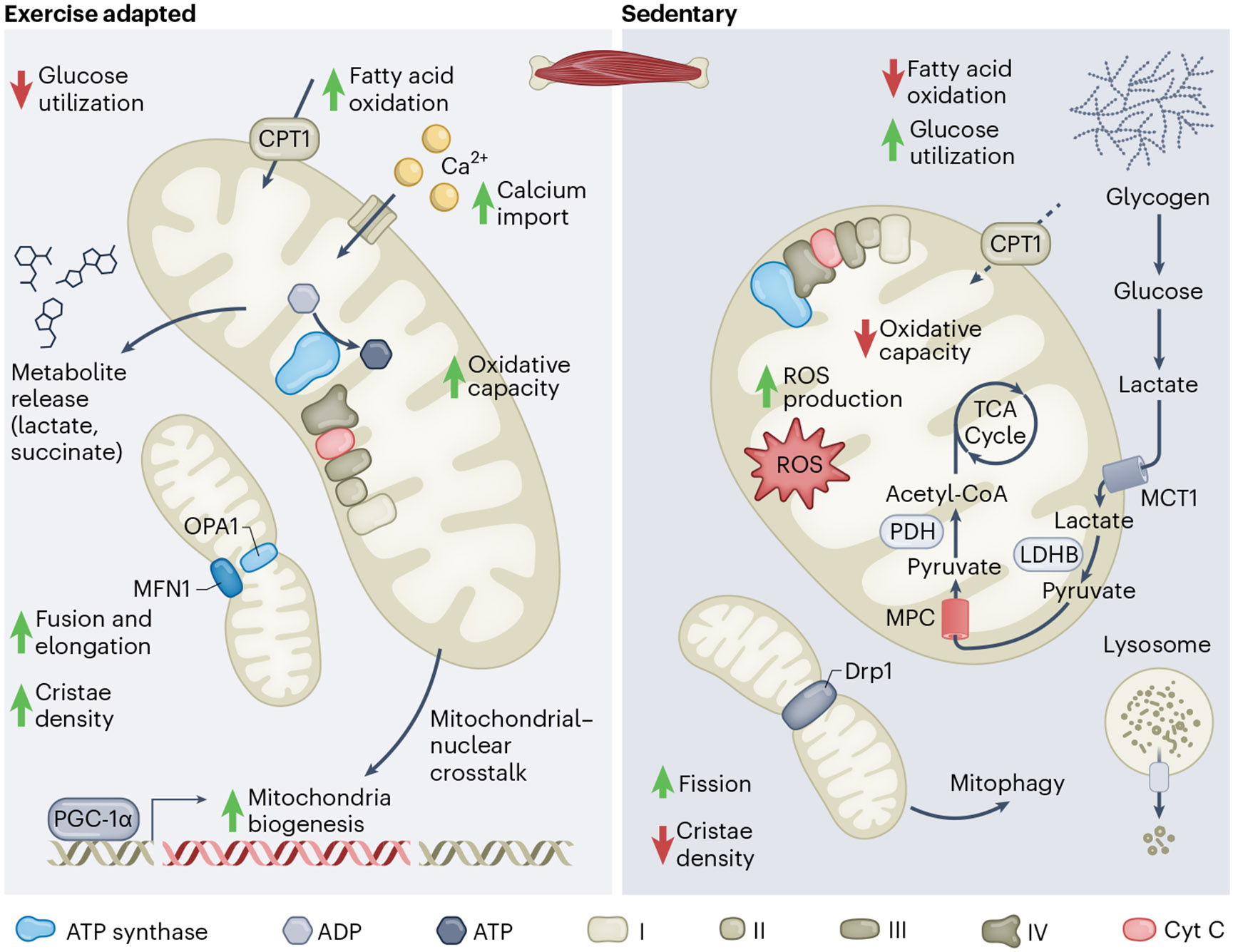
Exercise-adapted versus sedentary mitochondria. There is preferential reliance on OXPHOS activity and an increased propensity for fatty acid oxidation, underscored by increased expression of CPT1, in mitochondria that are in exercise-adapted skeletal muscle. As a result, there is reduced preference for anaerobic glycolysis and therefore, glucose utilization. Furthermore, more mitochondrial fusion driven by the proteins OPA1 and MFN1 occurs, which also increases cristae density. There is also increased Ca^2+^ import into the mitochondria. Mitochondrial–nuclear crosstalk results in increased mitochondrial biogenesis, which is in part driven by PGC-1α. Conversely, there is a strong preference for anaerobic glycolysis, as a result of rapid glycogen mobilization, in mitochondria in skeletal muscle of sedentary individuals. The glycogen-derived glucose is converted to lactate in the cytosol, which is transported into the intermembrane space via the action of monocarboxylate transporter 1 (MCT1). The lactate is converted to pyruvate via lactate dehydrogenase B (LDHB) in the IMS and the resulting pyruvate is transported into the matrix via the MPC. Pyruvate dehydrogenase (PDH) takes this pyruvate and feeds the TCA cycle via acetyl-CoA. There is reduced oxidative capacity and enhanced ROS production as well as decreased lipid oxidation capacity, in part driven by lower expression of CPT1. Enhanced fission results in degradation by mitophagy.

Mitochondrial function in skeletal muscle influences fibre adaptation during and after exercise, which contributes to fibre-type switching. Note that mitochondrial adaptation varies depending on either acute or long-term training and endurance aerobic exercise versus anaerobic exercise^86^. The two main types of skeletal muscle fibres, slow and fast twitch, differ in composition and function given the expression of myosin heavy chain type I, type IIA and type IIX, which correspond to the glycolytic or oxidative capacity of the specific fibre^87^. Owing to these differences, mitochondrial morphology and function vary between the two fibres, as reviewed elsewhere^88^. The well-known mitochondrial adaptations to exercise include mitochondrial biogenesis and increased capacity for oxidative ATP generation to fuel activity, among others, but further adaptations are emerging (Fig. 3). Mitochondrial succinate is released in response to exercise after a local pH change and potentiates skeletal muscle remodelling^89^. In addition, the GTP/GDP binding protein Rab8A promotes lipid droplet to mitochondria tethering in response to energy starvation conditions such as exercise to mediate the mobilization of long-chain fatty acids from lipid droplets to mitochondria^90^. Interestingly, subpopulations of mitochondria that are tethered to lipid droplets (peridroplet mitochondria) first described in brown fat have an elongated ultrastructure and low β-oxidative capacity, and are also enriched in type I fibres and the heart^91,92^. Proteomic and transcriptomic profiling revealed that components involved in mitochondrial respiration can be upregulated in trained muscle but are also enriched in muscle 8 h post exercise, suggesting an acute and chronic adaptation response^93^. Adaptations to exercise depend on the type, intensity and duration of exercise, which underscore the flexibility of mitochondria in relation to addressing cellular energy needs. However, beyond adaptation to exercise, particularly in situations of oxidative stress, muscle stem cells can fuse with existing myofibers and be removed from the stem cell compartment, and this process requires the actin reorganization protein Scinderin^94^. Induction of ETC damage via conditional knockout of ETC components likewise induces ROS and, subsequently, muscle stem cell fusion, removing ETC-dysfunctional muscle cells and impacting the repair of damaged muscle tissue^94^. As the effect of specific metabolites or changes in protein expression are being discovered, insight into the transcriptional regulatory processes that connect these adaptations to tissue function will be exciting to uncover.

How mitochondria induce fibre-type switching following energy demands is still an avenue of active research. For instance, skeletal muscle-specific deletion of carnitine palmitoyltransferase Ib (*Cpt1b*) impairs mitochondrial fatty acid oxidation but enhances mitochondrial biogenesis and increases the catabolism of amino acids as a major fuel source^95^. *Cpt2* deletion in skeletal muscle, which renders long-chain fatty acid oxidation defective, results in a switch towards glycolytic metabolism in oxidative tissues without affecting the expression of the myosin heavy chain isoform^96^. Through mitochondrial profiling, it was recently revealed that while long-chain fatty acids are an established fuel source during bouts of endurance exercise, mitochondria from slow fibre types are more lipid-tolerant than their counterparts derived from fast fibre types, and this tolerance is mediated by PGC-1α^97^. Furthermore, reverse flux of acetyl-CoA fuels activity of the enzyme medium-chain ketothiolase and enhances mitochondrial long-chain fatty acid tolerance by regenerating cofactors to support fatty acid oxidation activity^97^.

Furthermore, dysregulation of mitochondrial morphology via DRP1 depletion results in a defect in fast-twitch fibre differentiation without compromising respiratory capacity, signifying that mitochondrial ultrastructure acts in the determination of fast and slow-twitch fibres^98^. Deletion of the autophagy-regulating protein CREG1 in murine skeletal muscle reduces time to exhaustion, which was driven by accelerated mitophagy in the skeletal muscle^99^. Fibre types differ in mitochondrial length and rates of fusion and this led to the conclusion that the amounts of mitochondrial fusion directly correlate to oxidative capacity of the specific fibre^100^. However, in this study, mitochondrial morphology was not concluded to determine fibre type or contribute to fibre-type switching^100^. Mechanistically, the activation of the AMP-activated protein kinase (AMPK) pathway leads to phosphorylation of PGC-1α, the master regulator of mitochondrial biogenesis^101^. Notably, PGC-1α, which can increase fatty acid utilization, enhances oxidative slow-twitch gene expression, correlating mitochondrial biogenesis with fibre-type switching^102^. Interesting questions exist as to how to address the causality of fuel choice selection in response to exercise—if exercise-induced fuel choice selection is blocked selectively in the mitochondria, is it sufficient enough to affect fibre-type switching?

### The liver

**Fig. 4 ∣:**
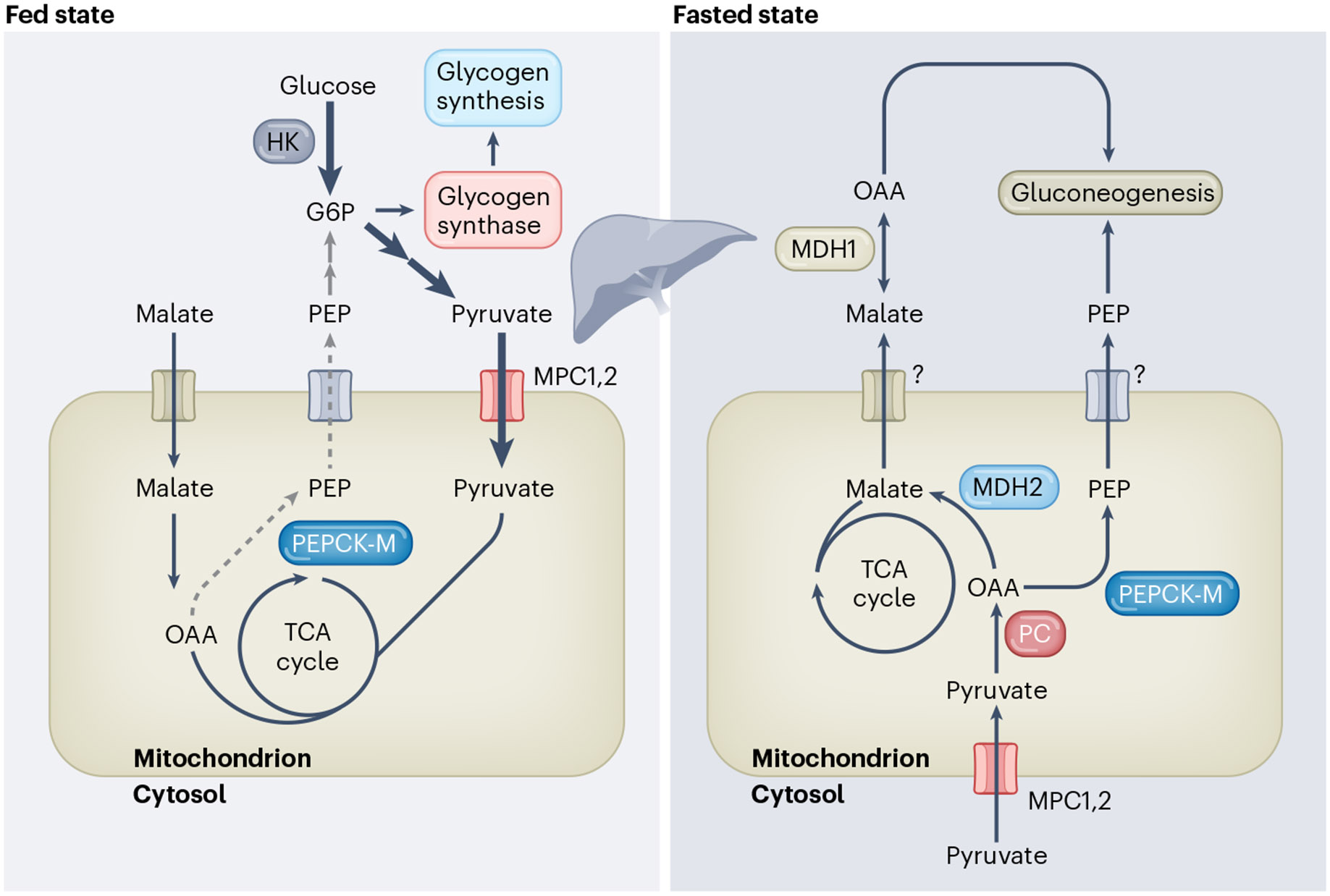
Mitochondrial fuel source selection in the fed versus fasted state. In the fed state, glucose is quickly converted to glucose-6-phosphate (G6P), demonstrated by the thick dark blue arrow to demonstrate fuel preference, which allosterically regulates glycogen synthase in the cytosol to promote glycogen synthesis. Malate is imported into mitochondria and converted to OAA, which fuels the TCA cycle. PEPCK-M activity is reduced in the fed state and the conversion of OAA to PEP is similarly reduced. PEP export out of the mitochondria, which would normally fuel glucose production, is reduced in the fed state, as demonstrated by the grey arrows. Glucose-derived pyruvate is transported into the mitochondria via the MPC in increased amounts, as demonstrated by the thick dark blue arrows. This pyruvate subsequently fuels TCA cycle activity. In the fasted state, glycogen-derived pyruvate is imported into mitochondria, which is converted to OAA by pyruvate carboxylase (PC). This OAA is converted to PEP via enhanced activity of PEPCK-M in response to fasting and the PEP is exported out of the mitochondria to fuel gluconeogenesis. OAA-derived malate is similarly exported out of the mitochondria.

In the liver, mitochondrial metabolite flux varies depending on the nutritional state. Serving as a hub that coordinates systemic metabolic processes, the liver is incredibly responsive to external cues such as stress, fasting, feeding and exercise^103^. Aerobic exercise decreases the number ofperidroplet mitochondria and lipid droplet size in the liver of mice fed a high-fat diet^104^. As the bulk of glucose production occurs in the liver, there is a constant flow of gluconeogenic substrates in and out of the liver that is governed by specific context-dependent cues, and substrate availability influences gene expression and protein function. For example, nocturnal animals that are calorie-restricted at night display higher levels of hepatic NADH during daytime, which subsequently inhibits SIRT1, drives a drop in body temperature, fatty acid oxidation and amino acid catabolism to conserve energy^105^. This is highly dependent on the NAD^+^/NADH ratio, as an expression of *Lb*NOX in the liver counteracts the effect of calorie restriction by increasing the concentration of liver metabolites and acylcarnitines in serum^105^. In the fed state, hepatic gluconeogenesis is decreased in the mitochondria and instead there is preferential activity of glycogen synthesis via the allosteric regulation of glycogen synthase by glucose-6-phosphate in the cytoplasm^106^. In addition, in the fed state, malate is imported into the mitochondrial matrix; however, in the fasted state, malate is exported out of the mitochondria and converted to OAA in response to activation of gluconeogenic enzymes by fasting-related hormones such as glucagon^107^ (Fig. 4). The key transcriptional changes in response to fasting and feeding are discussed elsewhere^108^. The expression of mitochondrial phosphoenolpyruvate carboxykinase (PEPCK-M), the mitochondrial isoform of PEPCK, is enhanced in the liver and contributes to hepatic gluconeogenesis^109^. Recent work showed that a liver-specific mitochondrial protein—the solute carrier SLC25A47—is required for exporting malate out of the mitochondria and loss of this protein results in decreased blood glucose^6,110^. Mitochondrial function is thus dependent on nutritional states: in the fed state, fuel source selection is mainly driven by the abundance of glucose-derived metabolites, which in turn fuel glycogen synthesis and the TCA cycle. However, in the fasted state, glycogen-derived pyruvate is converted to phosphoenolpyruvate (PEP) and other TCA-cycle-derived metabolites, which are exported out of the mitochondria to fuel gluconeogenesis.

The liver is a spatially heterogeneous organ with distinct zones that are determined by oxygen and metabolite flow as well as proximity to major vascular structures^111^. As periportal hepatocytes, which surround the hepatic artery and portal veins, are exposed to a higher oxygen partial pressure than pericentral hepatocytes, these cells tend to have a higher bioenergetic profile^112^. Periportal hepatocytes are enriched in glutathione and amino acid metabolic processes, marked by enriched expression of glutaminase 2 and histidine-ammonia lyase^113^. Furthermore, loss of the mitochondrial fatty acid oxidation enzyme long-chain acyl-CoA dehydrogenase induces steatosis in the periportal zone^114^. This zoning effect is mediated by SIRT5, which is a regulator of fatty acid oxidation in liver, as expression of SIRT5 is highly localized to the periportal region, and *Sirt5* deletion causes decreased fatty acid oxidation activity^114^. Pericentral hepatocytes, which surround the ventral vein, are enriched in lipid metabolic processes^115^. Furthermore, these hepatocytes are enriched in glutamine synthetase, as the ammonia byproduct of ureagenesis is converted to glutamine in pericentral hepatocytes^115^. Moving forward, profiling of the mitochondria within the distinct zones of the liver to reveal other adaptations, particularly in response to fasting and feeding, aside from the main bioenergetic properties and fuel choice selectivity, will be of interest. Furthermore, the existence of liver-specific mitochondrial proteins adds to the idea that mitochondria function in a context-dependent manner.

## Future directions

The ever-expanding roles of mitochondria in cellular physiology have changed our perception of these organelles. Mitochondria support the energy demands of a cell in response to external stimuli. However, it is also clear that mitochondria drive cell function, as manipulation of mitochondrial proteins that affect respiratory capacity or metabolite compartmentalization can alter behaviours such as fate determination and substrate selectivity. Many interesting questions remain. Although recent studies have expanded the list of mitochondrial protein functions, a number of mitochondrial proteins remain uncharacterized. In part, technical issues prevent the further study of these proteins; structural homology and predictive modelling do not necessarily reflect function. A prime example of this is UCP2–7; although UCP2–7 exhibit structural homology to UCP1, none of them seem to have thermogenic functionality^116^. In addition, in fungi, HEM25 is a homologue of mammalian SLC25A38, but the two proteins do not have the same identified function^117^. Some specific mitochondrial functions are cell-type- and time-dependent. SLC25A47 serves as an example of these parameters—its enrichment is dependent on nutritional states of the organism and its expression is localized to the liver^6^. New technologies to study organelle-specific flux will be of interest. Systems such as Mito-tag allow for rapid purification of mitochondria from a desired population; however, it still requires overexpression of an exogenous tag and in some cases purity may be compromised. Improvements in spatial metabolomic technologies will probably provide insight into both cell- and organelle-specific flux, as screening in a biologically relevant cell type in its native environment is of the utmost importance^118^.

The analysis of how signalling pathways sense a cell’s surroundings and the subsequent influence on mitochondrial behaviour is an area of active investigation; the application of fine-tuned metabolic assessments as well as specifically engineered systems to manipulate the cellular environment requires cooperation from many disciplines. These phenomena can translate to cell-specific alterations in mitochondrial function, as cellular environment varies depending on the location of the cell and can further vary depending on the pathophysiological state of the tissue—for example, stiff fibrotic tissue versus soft loose connective tissue such as adipose tissues. In addition, questions remain about mitochondrial adaptability in transient states of nutrient availability, such as during fasting or feeding, and subsequent fuel choice selection. Looking to the future, it will be important to continue to consider the state and context of the cell itself to perform specific assessments of mitochondrial composition and activities.

## Figures and Tables

**Table 1 ∣:** Cell- and tissue-specific changes of mitochondrial programmes and their functional results

Mitochondrial programme	Tissue or cell type	Functional implication	References
High MPC expression	NPCs	Maintenance of quiescence	Petrelli et al.^119^
Low OXPHOS expression	Liver	Reliance on fatty acid oxidation and glucose	McLaughlin et al.^76^
OXPHOS-dependent ATP production	Kidney	Localized to proximal tubule, where the majority of sodium filtering occurs	Tian and Liang^120^
Enrichment in cardiolipins	Muscle, brown adipose tissue	Supports lipid oxidative capacity	Kappler et al.^121^; Prola et al.^122^; Sustarsic et al.^123^
High acetyl-CoA usage	Regulatory T cells	Support interferon-γ production	Peng et al.^124^
High β-oxidation	Astrocytes	Fatty acid sink to protect neurons from fatty acid toxicity	Belanger et al.^79^; Ioannou et al.^78^
High β-oxidation	Cardiomyocytes	Consistent source of fatty acid substrate for ATP production	Cardoso et al.^125^; Schulze et al.^126^; Hui et al.^127^
Maximal CPT1 activity	Endurance-trained skeletal muscle	Preferential fatty acid usage for continuous fuel source	Fritzen et al.^85^; Smith et al.^87^
PEPCK-M expression	Liver	Supports hepatic gluconeogenesis	Mendez-Lucas et al.^109^
Tissue-specific expression of SLC25A47	Liver	Metabolite compartmentalization to support fasting gluconeogenesis	Yook et al.^6^; Bresciani et al.^110^
High MCU expression and Ca^2+^ uptake	Liver	Supports glucose metabolism and hepatocyte growth factor signalling	Paillard et al.^128^
High α-ketoglutaric acid accumulation	M2 macrophages	M2 macrophage polarization to resolve inflammation	Zhou at al.^129^; Liu et al.^130^
High succinate production	Bone marrow-derived macrophages	Succinate-dependent ROS production for the pro-inflammatory response	Mills et al.^131^
Low glutamine utilization	White adipose from obese individuals	Increased chromatin O-GlcNAcylation → expression of pro-inflammatory pathways	Petrus et al.^132^
Mitochondrial BCAA catabolism	Brown adipose tissue in cold exposure	Systemic insulin sensitivity	Yoneshiro et al.^133^; Yoneshiro at al.^134^
High iron–sulfur cluster biogenesis	Haematopoietic tissues	Support erythropoiesis and haeme synthesis	Galy et al.^135^
High proline synthesis	Pancreas	Supports protein synthesis	Tran et al.^136^; Ronn et al.^137^
High BCAA and BCKA metabolism	Heart	Supports cardiac protein synthesis over ATP production	Walejko et al.^138^
Glutamine production	Heart	Supports endothelial cell redox homeostasis	Murashige et al.^139^; Flam and Arany^140^; Durante^141^
Reliance on OXPHOS	Slow-twitch muscle fibres	Sustained energy production	Mishra et al.^100^; Muoio^142^
Higher mitofusin expression	Slow-twitch muscle fibres	Elongated mitochondrial ultrastructure for higher energy production	Mishra et al.^100^; Smith et al.^87^
High TCA cycle protein expression	Skeletal muscle	Generate reducing equivalents for ATP production	Kappler at al.^121^; Maurer et al.^143^
Low pyruvate carboxylase expression	Skeletal muscle	Pyruvate-derived complex I-dependent ATP production	Kappler et al.^121^
Increased Ca^2+^ uptake	Astrocytes	Motor and sensory output sensing	Li et al.^144^; Pickrell et al.^145^
Increased ACLY expression	White adipose tissue	Supports TCA cycle activity for ATP production and also de novo lipogenesis	Felix et al.^146^
UCP1 expression	Brown adipose tissue	Cold-induced thermogenesis	Kajimura et al.^147^

BCAA, branched-chain amino acid; BCKA, branched-chain keto acid; MCU, mitochondrial calcium uniporter.

## BOX 1 Overview of key mitochondrial pathways discussed

### Fatty acid oxidation.

β-Oxidation occurs primarily in mitochondria. For fatty acids in the cytosol to enter mitochondria, acyl-CoA synthetase adds a coenzyme (CoA) group to the fatty acid. The resulting fatty acid-CoA is transported into the mitochondria via the coordinated action of CPT1, CAC and CPT2. Per cycle of oxidation, β-oxidation generates one molecule of FADH_2_ and NADH, with the number of cycles and subsequent acetyl-CoA molecules produced depending on the length of the fatty acid. The resulting FADH_2_ and NADH molecules are used as reducing equivalents in the ETC and the acetyl-CoA molecules are fed into the TCA cycle.

### Glycolysis.

Occurring in the cytosol, glycolysis is an ATP and NAD^+^-dependent ten-step reaction that converts one glucose molecule into two pyruvate molecules, yielding two ATP and two NADH molecules in the process. The first step of the reaction (conversion of glucose to glucose-6-phosphate by hexokinase) is ATP-consuming, generating ADP as a byproduct. Glycolysis occurs very rapidly compared with other ATP-generating reactions such as OXPHOS.

### TCA cycle.

Although there are many inputs to the TCA cycle, one of the main starting points is the entry of pyruvate from the cytosol to the mitochondria via the MPC. As described above, acetyl-CoA generated from β-oxidation also feeds TCA cycle activity. A full cycle is completed when pyruvate is converted to OAA, generating one GTP molecule, three NADH molecules, one molecule of FADH_2_ and two CO_2_. The resulting NADH and FADH_2_ transfer electrons to the ETC. The cycle is allosterically regulated by NADH and ATP; accumulation of these molecules can shut off or taper activity. The TCA cycle also generates substrates for the ETC, such as succinate, which is expanded on in the section on OXPHOS.

### OXPHOS.

OXPHOS is characterized as the synthesis of ATP from ADP via a series of energetically favourable transfer of electrons from various electron donors and acceptors—that is, NADH, FADH_2_, coenzyme Q(10) and oxygen. NADH and FADH2 produced from glycolysis, the TCA cycle and β-oxidation are used during OXPHOS. Complex I (NADH dehydrogenase) and II (succinate dehydrogenase) turn over the TCA cycle substrates NADH and succinate to NAD^+^ and fumarate, respectively. Complex III (cytochrome c reductase) and IV (cytochrome c oxidase) operate via a series of electron transfer steps using cytochrome c as an electron donor and acceptor. Complex V (ATP synthase) activity is highly dependent on the proton gradient generated from the action of complexes I, III and IV as protons are pumped from the intermembrane space and, in the process, ADP and inorganic phosphate are converted to ATP.

### Generation of ROS.

Given that oxygen is utilized as an electron acceptor, the generation of ROS originating from mitochondria occurs during OXPHOS. This is primarily characterized by the transfer of one or two electrons to oxygen as a result of electron leakage mainly from complexes I and III, generating reactive superoxide or peroxide molecules. The resulting ROS cause damage to the mitochondria via oxidative stress, which causes homeostatic disruptions and even cell death.
